# The Role of 3D Printing in Pediatric Surgical Care: A Narrative Review

**DOI:** 10.7759/cureus.91839

**Published:** 2025-09-08

**Authors:** Jeremie Nallet, Olivier Hild, Yann Chaussy

**Affiliations:** 1 Orthopedic Surgery, Universite Besançon, Besançon, FRA; 2 Pediatric Surgery, CHU de Besançon, Besançon, FRA; 3 Pediatric Surgery, Université Marie et Louis Pasteur, Besançon, FRA

**Keywords:** 3d printing, pediatric surgery, preoperative planning, simulation in healthcare, systematic review (syst. rev.)

## Abstract

3D printing is rapidly expanding in surgery, with particular promise in pediatric surgery because of its ability to create customized objects tailored to individual needs. This study provides a comprehensive review of the literature on the main applications of 3D printing across pediatric surgical subspecialties. The review was conducted using the PubMed database. The search identified 207 articles, of which 96 were included after applying exclusion criteria. The most represented fields were orthopedics (n = 31) and head and neck surgery (n = 26). Reported applications of 3D printing included direct clinical use (n = 41), educational purposes (n = 28), surgical planning (n = 24), and assessment of model accuracy (n = 3). The key advantages of 3D printing include the production of anatomical models, both normal and pathological, for simulation, student education, surgical planning, and improved communication with families. Limitations include concerns about the realism of the models, cost, and difficulty in demonstrating clear clinical benefits. Comparative studies will be needed to objectively establish the value of 3D printing in pediatric surgery.

## Introduction and background

3D printing has been on the rise since the filing of a patent for the stereolithography technique in the 1980s. This technology, also called additive manufacturing or rapid prototyping, includes methods that create 3D objects by fusing or depositing successive layers of materials such as metals, polymers, or ceramics. Advances in this field, along with the development of high-performance and increasingly affordable 3D printers, have expanded its use across many areas, particularly in medicine.

In surgery, 3D objects can be created from 2D digital images, mainly CT scans and MRI, through several stages: image acquisition, image processing (segmentation and 3D model construction), pre-print processing, 3D printing, and post-print processing.

The potential of 3D printing is especially notable in pediatric surgery. Infants, children, and adolescents are constantly growing and undergoing morphological changes. 3D printing allows for the production of customized objects that meet each patient’s specific needs. It also enables the creation of models of rare pathologies, which are valuable for medical training and education.

The aim of this study is to review the main current applications of 3D printing in different pediatric surgical subspecialties and to discuss its advantages and limitations.

## Review

A narrative review of the medical literature was conducted using the PubMed database (National Center for Biotechnology Information, Bethesda, MD, USA) on September 22, 2023. The following search strategy was applied: (3D printing OR 3D printed) AND (Pediatric (Title/Abstract) OR Children (Title/Abstract)).

Articles were included if they were written in English and focused on the use of 3D printing in a pediatric surgical domain. Exclusion criteria were: general articles, articles not specifically addressing 3D printing, studies related to 3D printing of medications or bioprinting, articles unrelated to pediatric surgical fields, duplicate publications, studies related to dentistry/orthodontics, and reviews without original data.

The selected articles were categorized into four groups based on the domain of 3D printing utilization: simulation/education, studies using 3D printing for simulation and educational purposes in pediatric surgery, including anatomical models and surgical simulations for training medical professionals and students; surgical planning, studies applying 3D printing to surgical planning in pediatric cases, particularly through patient-specific 3D models to help surgeons understand anatomical complexities and plan procedures; clinical application, studies describing the direct clinical use of 3D printing in pediatric surgical procedures, including 3D-printed implants, surgical guides, and other patient-specific devices; and model evaluation, studies assessing the accuracy of 3D-printed models in pediatric surgery.

These categories were designed to systematically analyze and present the different applications of 3D printing across pediatric surgical subspecialties. Results are presented by subspecialty, followed by a discussion of the advantages/benefits and limitations/challenges of 3D printing in pediatric surgery.

Each included article was independently reviewed by two authors (JN and OH) to assess methodological quality and identify potential sources of bias. Discrepancies were resolved through discussion or, if necessary, consultation with a third reviewer (YC). For case reports and case series, evaluation criteria included clarity of patient demographics, relevant specialty, 3D printing applications, and reported advantages/benefits and limitations.

Results

**Figure 1:**
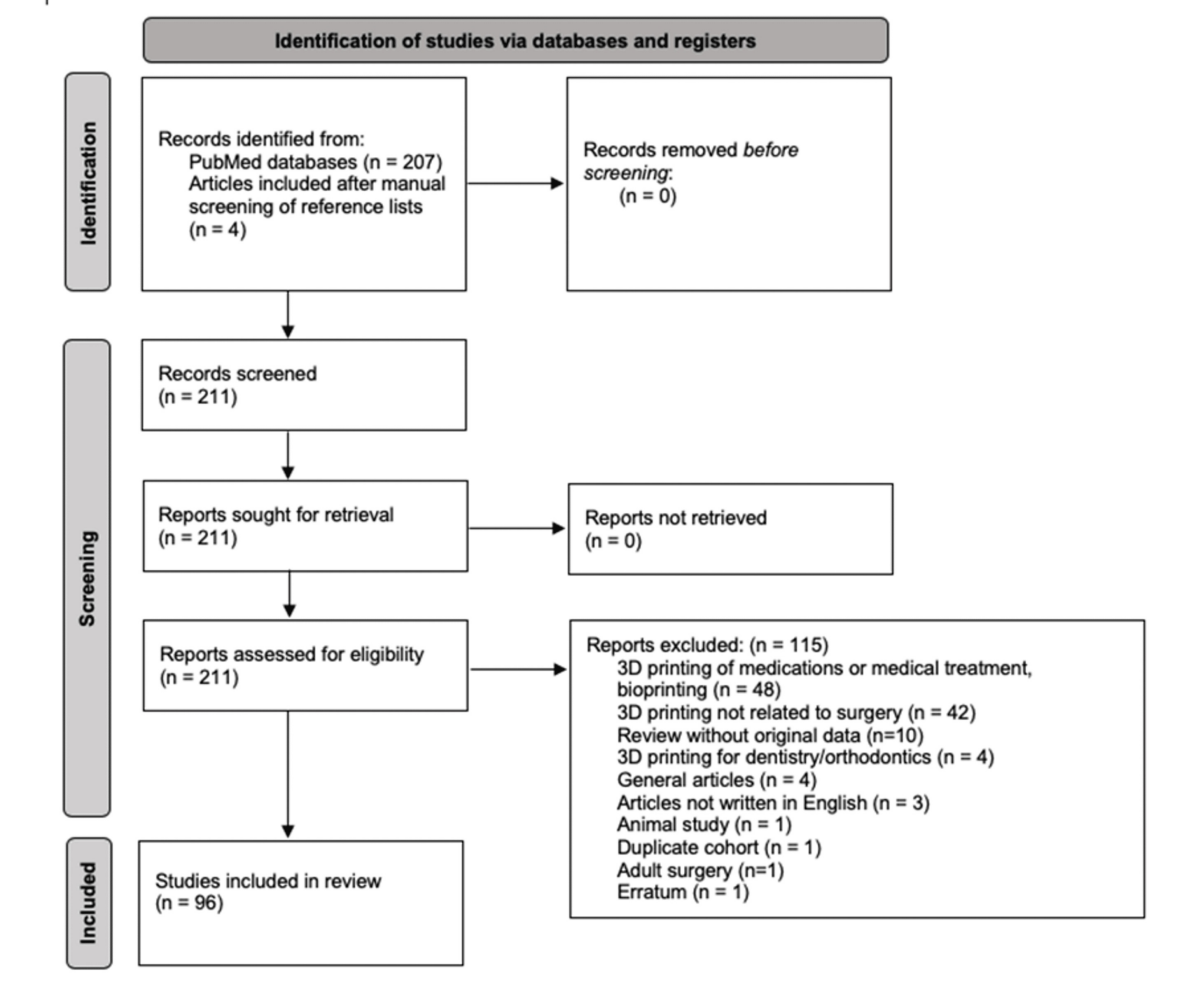
Flow diagram of the selected studies

The PubMed search identified 207 articles. Of these, 115 were excluded: 48 related to 3D printing of medications/bioprinting or medical treatment, 42 not related to any surgical domain, 10 reviews without original data, 4 related to dentistry/orthodontics, 4 general articles, 3 not written in English, 1 animal study, 1 duplicate article, 1 focused on adult surgery, and 1 erratum. In addition, four articles were identified through reference analysis of the included studies. Finally, 96 articles [1-96] were selected for this review on the use of 3D printing in pediatric surgery (Figure 1).

3D printing was reported in 28 articles for educational purposes (simulation sessions and student training), in 24 articles for surgical planning, in 41 articles for clinical applications, and in 3 articles for evaluating the accuracy of 3D-printed models (Table 1).

**Table 1:** Distribution of publications by pediatric surgical subspecialty and field of 3D printing application

Disciplines	Simulation/education	Surgical planning	Clinical application	Model evaluation
Orthopedics				
	Wickramasinghe et al. (2018) [1]; Caffrey et al. (2019) [2]; Neijhoft (2022) [3]	Cao et al. (2022) [5]; Facco et al. (2022) [6]; Bovid et al. (2019) [7]; Miao et al. (2024) [8]; Burzyńska et al. (2016) [9]	Zheng et al. (2017) [10]; Sun et al. (2023) [11]; Liu et al. (2022) [12]; Wan et al. (2021) [13]; Hu et al. (2020) [14]; Alessandri et al. (2022) [15]; Cai et al. (2023) [16]; Qu et al. (2023) [17]; Dow et al. (2022) [18]; Mohammadi et al. (2020) [19]; Ccorimanya et al. (2019) [20]; Gretsch et al. (2016) [21]; Zuniga et al. (2018) [22]; Zuniga et al. (2015) [23]; Zuniga et al. (2019) [24]; Bhat et al. (2021) [25]; Zuniga et al. (2019) [26]; Cabibihan et al. (2020) [27]; Lazzeri et al. (2022) [28]; Katt et al. (2021) [29]; Elbaum (2020) [30]; Wojciechowski et al. (2022) [31]	Hedelin et al. (2019) [4]
Head and neck surgery (ENT, maxillofacial surgery, ophthalmology)				
	Lioufas et al. (2016) [32]; Chou et al. (2017) [33]; Jimenez et al. (2023) [34]; Longfield et al. (2015) [35]; Freiser et al. (2021) [36]; Barber et al. (2016) [37]; Kavanagh et al. (2017) [38]; Chebib et al. (2022) [39]; Reighard et al. (2019) [40]; Falls et al. (2022) [41]; Michaels et al. (2022) [42]; Chang et al. (2020) [43]	Lee et al. (2019) [44]; Rose et al. (2015) [45]; Arcieri et al. (2018) [46]	Morrison et al. (2015) [47]; Les et al. (2019) [48]; Ramaraju et al. (2022) [49]; Wang and Qi (2020) [50]; Akiki et al. (2021) [51]; Chai et al. (2021) [52]; Arunkumar et al. (2023) [53]; Wu and Lv (2023) [54]; Thurzo et al. (2022) [55]; Groot et al. (2023) [56]; Di-Luciano et al. (2022) [57]	
Neurosurgery and spinal surgery				
	London et al. (2021) [58]; Weinstock et al. (2017) [59]; González-López et al. (2023) [60]; Graffeo et al. (2023) [61]	Weinstock et al. (2015) [63]; Cao et al. (2021) [64]; Vissarionov et al. (2020) [65]	Lou et al. (2022) [66]	Chacko et al. (2023) [62]
Cardiac and vascular surgery				
	Hopfner et al. (2021) [67]; Brunner et al. (2022) [68]; Awori et al. (2023) [69]; Karsenty et al. (2022) [70]	Pizzuto et al. (2022) [71]; Ghosh et al. (2022) [72]; Ryan et al. (2018) [73]		Parthasarathy et al. (2021) [74]
Oncological surgery				
	Youn et al. (2023) [78]	Riggs et al. (2018) [75]; Jug (2021) [76]; Parthasarathy et al. (2023) [77]; Youn et al. (2023) [78]; Girón-Vallejo et al. (2018) [79]; Sánchez-Sánchez et al. (2018) [80]	Beltrami et al. (2021) [81]; Gong et al. (2023) [82]; Zhu et al. (2021) [83]	
Digestive surgery				
	Burdall et al. (2016) [84]; Williams et al. (2018) [85]	Park et al. (2022) [86]; Krois et al. (2022) [87]		
Thoracic surgery				
	Hong et al. (2021) [88]	Matsuo et al. (2018) [89]	Duan et al. (2022) [90]	
Urology				
	Lemarteleur et al. (2021) [91]	Chandak et al. (2019) [92]	He et al. (2022) [93]	
Plastic surgery/burns				
			Wei et al. (2017) [94]; Şenaylı et al. (2021) [95]	
Gynecology				
	Crain et al. (2021) [96]			

**Figure 2:**
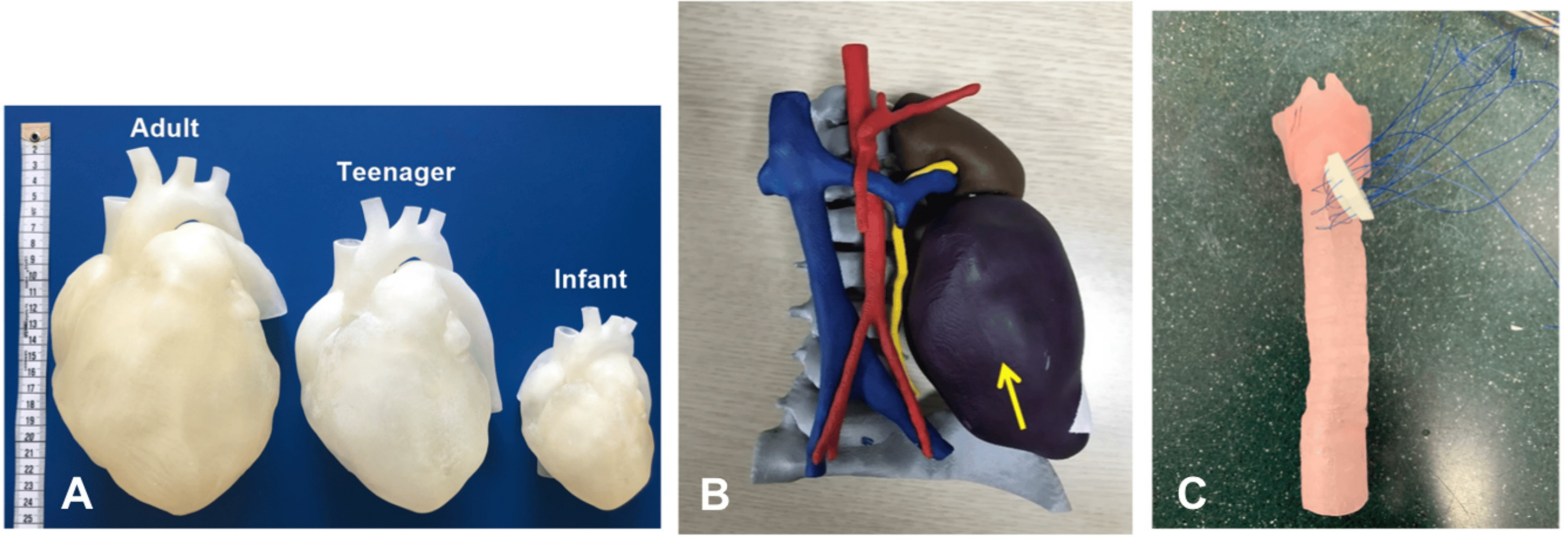
Examples of 3D-printed models for simulation or education (A) 3D-printed heart models in different sizes (Source: [68]). (B) 3D-printed heart model of a four-year-old male patient with neuroblastoma (yellow arrow) (Source: [78]). (C) Simulation of an anterior approach to airway reconstruction on a 3D-printed model (Source: [41]). All images are taken from open-access articles licensed under the Creative Commons Attribution 4.0 International License, permitting use without modification or commercial purposes (http://creativecommons.org/licenses/by/4.0/).

**Figure 3:**
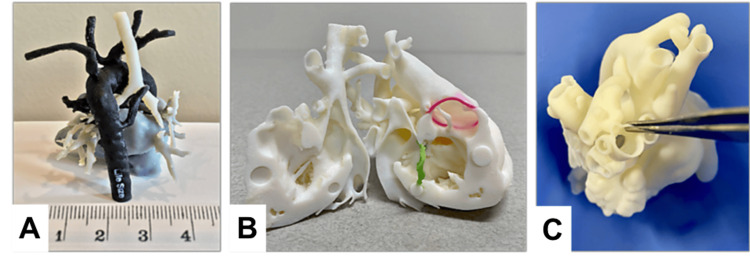
Examples of 3D-printed models for surgical planning Patient-specific models demonstrating: (A) Tetralogy of Fallot with pulmonary atresia and major aortopulmonary collaterals, (B) anatomy of the right ventricle to aorta (pink = aortic annulus, green = tricuspid annulus), (C) thickness of the vessel wall. (Source: [72] (Additional file 2: Supplemental Fig. 2)). All images are taken from an open-access article licensed under the Creative Commons Attribution 4.0 International License, permitting use without modification or commercial purposes (http://creativecommons.org/licenses/by/4.0/).

**Figure 4:**
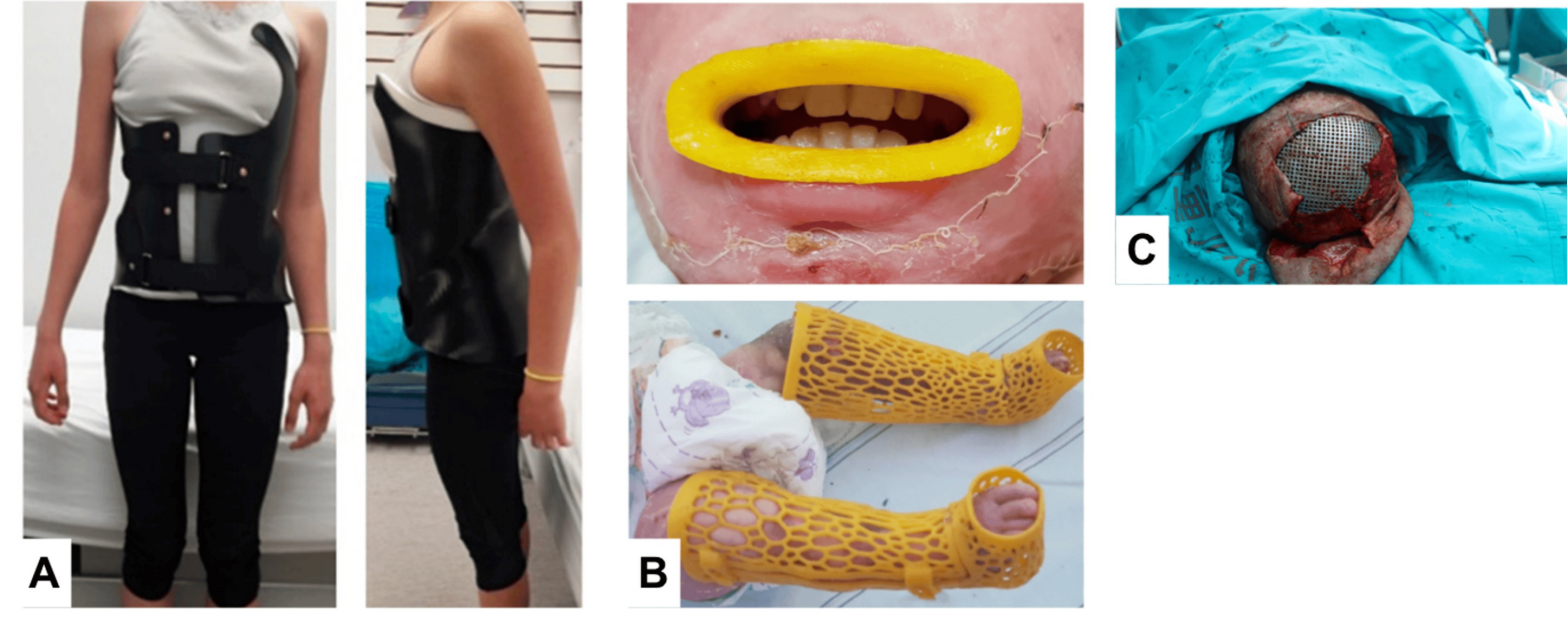
Examples of 3D-printed models for clinical use (A) 3D-printed spinal brace for scoliosis (Source: [66]). (B) 3D-printed splints to avoid contracture development in burned children (Source: [95]). (C) 3D-printed titanium mesh to repair skull defect (Source: [50]). All images are taken from open-access articles licensed under a Creative Commons Attribution 4.0 International License, permitting their use, without any change or commercial use (http://creativecommons.org/licenses/by/4.0/).

Examples of 3D-printed models used for simulation/education, preoperative planning, and clinical applications are shown in Figures 2-4, respectively.

The most represented disciplines in the selected articles were orthopedics (n = 31) and head and neck surgery (n = 26). Other applications of 3D printing were reported in neurosurgery and spinal surgery (n = 9), cardiac and vascular surgery (n = 8), oncological surgery (n = 9), digestive surgery (n = 4), thoracic surgery (n = 3), urology (n = 3), plastic surgery (n = 2), and gynecology (n = 1).

Current applications of 3D printing in various pediatric surgical subspecialties are provided in Table 2.

**Table 2:** Current applications of 3D printing in various pediatric surgical subspecialties 3DP: 3D-printed.

Simulation/education	Surgical planning	Clinical application
Pediatric orthopedics		
3DP models to compare surgical approaches in pediatric pelvic osteotomies and for hands-on teaching courses of pediatric traumatology (ankle fracture)	Preoperative measurement and surgical planning of patients with developmental hip dysplasia; preoperative planning of supracondylar humerus osteotomies for correction of cubitus varus; preoperative preparation of calcaneal fractures treatment; planning of lower limb lengthening and axis correction surgery	3DP navigation templates for correction of developmental dysplasia of the hip; 3DP cutting guides for the treatment of cubitus varus, correction of severe genu varum, and resection of lower limb epiphyseal complex lesions; 3DP prostheses, especially hand prostheses; 3DP patient-specific casts or orthoses for upper extremity fractures, clubfoot, or ankle-foot disorders in Charcot-Marie-Tooth disease; 3DP mold to create a personalized cement spacer to treat periarticular infection; 3DP titanium cage in lower limb salvage surgery
Pediatric head and neck surgery (ENT, maxillofacial surgery, ophthalmology)		
3DP models of cleft lip and palate for surgical education and improving parental information; 3DP models for endoscopic endonasal skull base surgery; 3DP pediatric temporal bones for mastoidectomy simulation; 3DP simulators for endoscopic ear surgery or laryngoscopy	Surgical planning of complex cases: juvenile aggressive ossifying fibroma, recurrent cholesteatoma, or congenital tracheal stenosis associated to pulmonary sling	3DP patient-specific airway splints to treat severe tracheobronchomalacia; 3DP models for complex reconstruction of craniofacial region, orbital floor reconstruction, treatment of familial gigantiform cementoma, management of multiple mandibular fractures, or cranio-facial syndromes like Pierre Robin sequence; 3DP conformers in congenital microphthalmia and anophthalmia
Pediatric neurosurgery and spinal surgery		
3DP model of pediatric skull base for minimally invasive endonasal approaches; 3DP model of hydrocephalus for endoscopic third ventriculostomy training; 3DP models of rare pediatric cerebrovascular diseases like aneurysms, arteriovenous malformations, and vein of Galen malformations	Preoperative planning of cerebrovascular surgical and endovascular procedures	3DP navigation templates for pedicle screw placement in the treatment of pediatric congenital scoliosis; 3DP spinal braces
Pediatric cardiac and vascular surgery		
3DP models for hands-on catheter workshops in pediatric cardiology; 3DP models of congenital heart disease for surgical education and parental information	Preoperative planning of cardiothoracic surgery and catheter-based interventions for patients with acquired or congenital heart disease	
Pediatric oncological surgery		
	3DP model of cardiac tumor to preoperatively plan tumor debulking; 3DP model to plan screw placement after a cervical spine tumor resection; 3DP model for complex surgery of osteo-articular tumors (Ewing’s sarcoma, osteosarcoma, and chondrosarcoma); 3DP model to plan retroperitoneal tumors resection (neuroblastoma, ganglioneuroma); 3DP model to plan nephron-sparing surgery in bilateral Wilms tumor; 3DP model to plan surgery in complex solid tumors cases (nephroblastoma, neuroblastoma, pulmonary metastases)	3DP custom-made implants for the reconstruction of bony defects after skeletal tumors resection
Pediatric digestive surgery		
3DP model for laparoscopic choledochal surgery training; 3DP model for laparoscopic pyloromyotomy training	3DP model of abdominal cavity of liver transplantation recipient to prevent large-for-size syndrome; 3DP model of cloacal malformation for preoperative planning	
Pediatric thoracic surgery		
3DP phantoms for thoracoscopic esophageal atresia surgery training	3DP model for surgical planning of Nuss procedure (pectus excavatum)	3DP patient-specific bronchial blocker for infants
Pediatric urology		
3DP model of upper urinary tract for laparoscopic pyeloplasty training	3DP patient-specific model for preoperative planning of complex living-donor pediatric renal transplantation	3DP extravascular stent for nutcracker syndrome
Pediatric plastic surgery (burn surgery)		
		3DP facemasks to treat facial hypertrophic scars after burns; 3DP splints to avoid contracture development in burned children
Pediatric gynecology		
3DP vulvar model to teach pediatric straddle injury repair		

Pediatric orthopedics

Orthopedic and trauma surgery was the most represented specialty.

Only two articles described the use of 3D printing for simulation or educational purposes. Caffrey et al. [2] used 3D-printed models to compare surgical approaches in pediatric pelvic osteotomies. They evaluated changes in acetabular morphology created by three osteotomy techniques using matched patient-specific 3D-printed pelvic models. Neijhoft et al. [3] created 3D-printed models of transitional ankle fractures, which were used in hands-on teaching courses in pediatric traumatology. These models helped improve the 3D understanding of fractures that are often difficult to grasp with traditional 2D educational materials.

As with all diagnostic and therapeutic methods in medicine, quality assessment of 3D-printed models is necessary to ensure reliable performance. Hedelin et al. [4] presented an image-based strategy for assessing the quality of 3D-printed pelvic models using locally available diagnostic tools. They reported a strong linear correlation between the models and ground-truth images both preoperatively and postoperatively.

Several articles highlighted the value of 3D printing for surgical planning, particularly in correcting complex orthopedic conditions. For example, 3D printing has been applied in preoperative measurement and planning for patients with developmental hip dysplasia [5,6]. Cao et al. [5] compared outcomes of proximal femoral osteotomy in 20 patients who underwent preoperative planning with 3D-printed models versus 20 patients planned using standard methods without 3D printing. While clinical efficacy, evaluated by McKay’s criteria, was similar in both groups, the 3D printing group showed shorter operation times, reduced intraoperative blood loss, and fewer X-ray fluoroscopies. 3D modeling and printing have also been used in the preoperative planning of supracondylar humerus osteotomies for correction of cubitus varus, a challenging complication following pediatric elbow fractures [7]. Practicing the correction on 3D-printed models was particularly useful for planning the rotational component of the procedure. Additionally, 3D-printed models have been applied in the preoperative preparation of calcaneal fracture treatment in children [8] and in planning lower limb lengthening and axis correction surgery in a young child using the Ilizarov method [9].

A clinical application can complement surgical planning when 3D printing is used to create specific cutting guides for intraoperative use. 3D-printed navigation templates have been developed for proximal femoral osteotomy [10,11] and Tönnis triple osteotomy [12] in children with developmental dysplasia of the hip. In these studies, preoperative 3D-printed navigation templates helped reduce operative time, radiation exposure, and intraoperative blood loss. However, clinical efficacy was similar between groups according to McKay’s and Severin’s criteria when surgery was performed by experienced surgeons. Patient-specific 3D-printed cutting guides have also been reported for the treatment of cubitus varus [13,14], correction of severe genu varum [15], and resection of lower limb epiphyseal complex lesions [16], with favorable outcomes. Other orthopedic applications include a mold for producing personalized cement spacers to treat periarticular infection after bone tumor resection [17], and a titanium cage for lower limb salvage surgery following traumatic partial amputation of the ankle [18]. Another expanding clinical application is the production of prostheses, particularly hand prostheses for children with congenital or traumatic upper limb amputations [19,24]. These 3D-printed transitional prostheses are inexpensive, customizable to the patient’s needs and limb deficiency, easily replaceable as the child grows, and useful in improving grasping activities [25]. Zuniga et al. [26] described a method for remotely fitting 3D-printed upper limb prostheses using only patient photographs to extract anthropometric measurements. In war-affected and low-resource countries, 3D-printed prosthetic hands offer additional benefits such as portable printers, cost-effective materials, on-site production, amputee-specific design, and low maintenance costs [27]. Further studies are required to assess the long-term benefits, durability, and rejection rates of these low-cost devices. Finally, 3D printing has been used for patient-specific casts and orthoses in the management of upper extremity fractures [28,30], clubfoot [30], and ankle-foot disorders in Charcot-Marie-Tooth disease [31]. The 3D anatomy acquisition phase can be performed with an optical scanner, thereby avoiding radiation exposure. Compared with traditional plaster casts, 3D-printed casts were lighter, provided ventilation, and allowed visualization of the underlying skin. However, their use requires production time and a team trained in 3D printing technology.

Pediatric head and neck surgery (ENT, maxillofacial surgery, and ophthalmology)

Several authors reported the use of 3D-printed models for simulation. Lioufas et al. [32] developed three 3D-printed cleft lip and palate models for surgical education and also highlighted their value in improving parental understanding. This benefit was supported by Chou et al. [33], who involved 30 parents of children with cleft lip and palate and found that the models improved parental information. Jimenez et al. [34] used 3D-printed models for preoperative parental and patient counseling before endoscopic endonasal skull base surgery in children. They concluded that parents and patients older than four years considered the models helpful for understanding their condition and planned surgery. Other authors reported the use of 3D-printed pediatric temporal bones for mastoidectomy simulation, which could be produced quickly and at low cost [35,36]. Similarly, 3D printing was used to fabricate simulators for endoscopic ear surgery [37] and laryngoscopy [38]. Tracheobronchial tree models were created for training in foreign body extraction [39] and for laryngotracheal reconstruction in subglottic stenosis [40]. Falls et al. [41] compared the educational value of 3D-printed models and porcine cadaveric models for laryngotracheal reconstruction simulation. Both were rated similarly for overall training utility, surgical planning, and improving operative techniques, but the 3D-printed model was rated more useful for teaching anatomy. The development of 3D-printed simulators also facilitated the implementation of national simulation programs, particularly in pediatric otolaryngology fellowship training [42,43]. These virtual surgical dissection courses showed that 3D-printed simulators were effective in teaching advanced surgical techniques in a low-risk environment. Virtual platforms also proved to be a viable and well-rated option for surgical training when in-person teaching was restricted, such as during the COVID-19 pandemic.

3D printing was also applied in surgical planning [44], particularly for complex cases such as the management of a juvenile aggressive ossifying fibroma, a planned canal-wall-down tympano-mastoidectomy for recurrent cholesteatoma [45], or congenital tracheal stenosis associated with a pulmonary sling [46]. Accurate 3D models can be rapidly generated from clinical CT scans to simulate challenging pediatric cases preoperatively, potentially reducing medical errors and improving patient safety. A key advantage of 3D printing is the ability to visualize and physically handle a model that replicates the operative field, allowing surgeons to manipulate it or rehearse the planned technique.

Beyond planning, 3D printing has direct clinical applications through the design and production of implantable and non-implantable medical devices. In airway surgery, it has been particularly valuable for children with tracheobronchomalacia. A team from Michigan reported their experience designing and producing patient-specific, bioresorbable airway splints in 15 critically ill children with severe tracheobronchomalacia [47,48]. These external splints, manufactured from polycaprolactone (a biocompatible, bioresorbable polyester), remain in vivo for 2-3 years before resorption. All surviving patients experienced significant clinical improvement, and no patient required splint removal or reoperation. The authors also introduced the concept of 4D printing, in which 3D-printed devices are designed to change shape over time in response to growth and resorption, eliminating the need for surgical removal. Ramaraju et al. [49] described the complete process of developing a 3D-printed implantable device, from conception through validation to clinical application. Other reported uses include reconstruction of a child’s craniofacial region [50], orbital floor reconstruction [51,52], treatment of familial gigantiform cementoma with facial skeleton reconstruction [53], management of multiple mandibular fractures [54], and creation of personalized biomedical appliances for children with craniofacial syndromes such as Pierre Robin sequence [55].

In ophthalmology, 3D-printed conformers were used for socket expansion in congenital microphthalmia and anophthalmia [56]. Di-Luciano et al. [57] also designed a 3D-printed trocar spacer that shortened the length of the trocar for pediatric vitreoretinal surgery.

Pediatric neurosurgery and spinal surgery

Several studies have reported the use of 3D printing for neurosurgical simulation in pediatrics. London et al. [58] developed a 3D-printed model of the pediatric skull base to assess its value as a training tool for minimally invasive endonasal approaches. Weinstock et al. [59] produced a highly realistic hydrocephalus model by combining 3D printing with special effects techniques, validating its use in training fellows and residents to perform endoscopic third ventriculostomy. González-López et al. [60] designed a simpler 3D-printed hydrocephalus model aimed at training neurosurgeons without prior endoscopic experience, particularly in low-resource settings. Training with this model led to significant improvements in procedure time, number of fenestration attempts, fenestration diameter, and avoidance of critical structures. Graffeo et al. [61] described a reproducible set of 3D models for optimizing training in rare pediatric cerebrovascular diseases such as aneurysms, arteriovenous malformations, and vein of Galen malformations. To support broader educational use, they also provided open-source resources and templates for model reproduction and dissemination.

Chacko et al. [62] evaluated the size fidelity of 3D-printed brain models derived from MRI scans of children with prior hypoxic-ischemic injury. They confirmed that 1:1 scale models preserved accurate linear measurements and could therefore be reliably used for medical training and clinical consultation.

3D printing has also been applied in surgical planning, including the optimization of cerebrovascular and endovascular procedures in children [63]. Several studies reported that 3D-printed navigation templates improve the accuracy and safety of pedicle screw placement in pediatric congenital scoliosis compared with the freehand method [64,65]. While image-guided navigation systems and robotic-assisted screw placement achieve higher accuracy, they are costly, require technical expertise, and increase intraoperative radiation. In contrast, 3D printing offers a practical alternative to mitigate these limitations [64]. However, comparative studies between 3D-printed navigation templates, image-guided navigation, and robotic screw placement have not yet been reported.

Beyond intraoperative applications, 3D printing has also been explored in orthotics. One study demonstrated that 3D-printed spinal braces were 33% thinner, 26% lighter, 37% less expensive, and required 3.7 fewer labor hours to manufacture compared with standard polypropylene braces [66].

Pediatric cardiac and vascular surgery

3D printing has been applied in pediatric cardiology to create educational models for anatomy courses and training models for hands-on catheter workshops [67,68]. Patient-specific models derived from CT or MRI images were digitally modified to represent various cardiac pathologies. This approach allowed the production of teaching models independent of the incidental availability of patient data. However, the use of digital modification techniques carries the risk of generating unrealistic anatomical features, particularly when performed by engineers without medical training [67]. Awori et al. [69] compared the educational value of Virtual Reality (VR) and 3D-printed models for teaching residents and nurse practitioners about a normal heart and tetralogy of Fallot. While both modalities enhanced understanding, participants reported greater clarity with VR models, with 87% preferring VR over 3D printing. In contrast, Karsenty et al. [70] assessed the role of 3D-printed cardiac models in parental counseling before interventional catheterization. They found that the models improved parental understanding of the procedure and reduced anxiety levels.

Pizzuto et al. [71] emphasized that pediatric cardiothoracic surgery and catheter-based interventions require a precise understanding of complex and heterogeneous anatomy. By improving visuospatial comprehension of patient-specific cardiac lesions, 3D representations were shown to be valuable adjuncts to conventional imaging, adding benefit to preoperative planning. In a retrospective study spanning 2018-2020, Ghosh et al. [72] described the integration of a clinical 3D modeling service into routine pre-procedural care for 112 pediatric patients with acquired or congenital heart disease. During the first year, cases were primarily presented as printed models with 3D PDFs for flat-screen viewing. Over the following two years, there was a shift toward newer visualization formats, including computer-aided design (CAD)-based modeling of potential surgical repairs and virtual reality model viewing, accompanied by a decrease in the reliance on printed models. Ryan et al. [73] compared standard-of-care pre-procedural planning with 3D printing-assisted planning to evaluate the impact of anatomical models. While statistical significance was not demonstrated using ANOVA and Fisher’s exact test, trends indicated shorter operating room times and overall case durations in the 3D printing group. Moreover, surgeons surveyed using the technology acceptance model reported that 3D-printed models were effective tools for planning complex congenital heart disease repairs.

Parthasarathy et al. [74] evaluated the accuracy of patient-specific 3D-printed models derived from CT angiography in children with anomalous aortic origin of a coronary artery. They compared the CT scans of the printed models with the patients’ segmented CT data and found that the models accurately represented the pathological anatomy. These models may facilitate hemodynamic flow evaluation, refinement of risk stratification criteria, patient education, and therapeutic decision-making.

Pediatric oncological surgery

3D-printed models have been described for oncologic surgical planning. For example, 3D-printed cardiac tumor and anatomical models were used to plan two pediatric tumor debulking procedures, helping devise safe strategies for maximal tumor removal while protecting the coronary circulation [75]. A 3D-printed model also aided screw placement after cervical spine tumor resection in a 4-year-old child [76-79]. Parthasarathy et al. [77] detailed the complete workflow for creating 3D anatomical models in 12 complex tumors (5 Ewing’s sarcomas, 6 osteosarcomas, and 1 chondrosarcoma), from image acquisition to printing. This approach provided a cost-effective tool for preoperative planning and was regarded by surgeons as a powerful means of communication with patients and families, particularly when discussing surgical risks and obtaining informed consent. Similarly, Youn et al. [78] evaluated 3D-printed models in 10 pediatric retroperitoneal tumor resections. They found that the models improved surgeons’ understanding of the anatomy, enhanced students’ learning, and helped guardians better comprehend the clinical scenario during informed consent.

3D-printed models have also been used for the surgical planning of complex solid tumors. Girón-Vallejo et al. [79] reported that a 3D-printed model of bilateral Wilms tumors improved understanding of the anatomical relationship between the tumors and the normal kidneys, thereby enhancing surgical planning and preoperative discussions with the patient’s family. Similarly, Sánchez-Sánchez et al. [80] applied 3D printing in four complex oncological cases (bilateral Wilms tumor, bilateral pulmonary metastases, abdominal neuroblastoma, and cervico-thoracic neuroblastoma). They found that 3D reconstructions and full-scale printed models were valuable tools in planning tumor resections, as they clarified the relationship between tumors and adjacent organs and helped anticipate potential surgical complications. These models also improved family understanding, thereby strengthening doctor-patient communication.

Clinical applications of 3D printing have also been reported in oncological orthopedic surgery. Beltrami et al. [81] described a series of 11 pediatric patients with skeletal tumors (6 Ewing’s sarcoma, 4 osteosarcoma, and 1 rhabdomyosarcoma) treated with custom-made implants for reconstruction of bony defects. The osteotomy level was determined by the surgeon, and the implant was then designed to match the segmental gap after tumor removal. The implant design incorporated surgeon-specified features to ensure secure fixation to the host bone. Gong et al. [82] reported a joint-sparing surgical technique for distal femoral malignancies, using intraoperative physeal distraction combined with a 3D-printed endoprosthesis in seven pediatric patients. Zhu et al. [83] described the use of customized 3D-printed prostheses to reconstruct the acetabulum in two children with periacetabular Ewing’s sarcoma. Under virtual conditions, tumor excision with safe margins was simulated, and a prosthesis was designed to perfectly fill the defect. The design process required about two days, and printing, packaging, and shipping took an additional three to four days, resulting in a total lead time of approximately one week from request to implant availability.

Pediatric digestive surgery

3D printing has been used to simulate laparoscopic choledochal surgery [84] and laparoscopic pyloromyotomy [85].

For surgical planning, 3D-printed models of the abdominal cavity of liver transplantation recipients were created to help prevent large-for-size syndrome [86]. Large grafts can lead to difficult abdominal closure, graft compression, reduced oxygen supply, and subsequent graft dysfunction. The 3D-printed abdominal cavity model was produced in less than 10 hours at minimal cost and proved useful for preventing large-for-size syndrome in small recipients, especially pediatric patients. However, the authors noted that the model did not reproduce the actual elasticity of the abdominal wall and diaphragm. Krois et al. [87] also described a 3D-printed model of an infant pelvis with a cloacal malformation for preoperative planning and training. Their findings suggested that patient-specific 3D-printed models may be a valuable tool in the preoperative evaluation of complex anorectal malformations, allowing simulation of cysto-vaginoscopy with excellent visualization of anatomical structures.

Pediatric thoracic surgery

For simulation and education, 3D-printed phantoms for video-assisted thoracoscopic surgery (VATS) were created from chest CT data of a pediatric patient with esophageal atresia and tracheoesophageal fistula [88].

Patient-specific 3D-printed models have also been used for surgical planning of the Nuss procedure, helping to determine the optimal number and placement of bars to achieve the best outcome and to predict the degree of improvement [89].

3D printing can further support the design of medical devices or consumables tailored to special populations such as infants. Duan et al. [90] reported the use of 3D printing to design a new bronchial blocker for infants, as commercially available devices do not adequately match their anatomical characteristics.

Pediatric urology

Lemarteleur et al. [91] designed and printed a low-cost, environmentally friendly simulator of the upper urinary tract with anatomical variants requiring complex procedures such as pyeloplasty. The model had two components: a reusable part representing the parenchyma and a single-use part for the urinary tract. Survey results showed that the simulator met most expectations for laparoscopic pyeloplasty training. However, face validity assessment highlighted a lack of material elasticity during the procedure.

Chandak et al. [92] explored a novel use of patient-specific 3D printing to improve preoperative planning in complex living-donor pediatric renal transplantation. They applied the technique in three situations: (1) significant size discrepancy between an adult donor kidney and a child recipient’s abdomen, (2) vascular anomalies, and (3) staged operations for aneurysmal disease. The authors concluded that 3D models provided critical insights into intra-abdominal space and optimal anastomotic windows, especially in the presence of vascular anomalies. They also improved communication with patients and families. Reported limitations included the inability of 3D models to reproduce human tissue properties, the need for specialized expertise, and cost.

A clinical application was also described involving a 3D-printed extravascular stent to treat a 14-year-old boy with nutcracker syndrome. The stent was designed to elevate the superior mesenteric artery and lower the duodenum, thereby relieving compression on the left renal vein [93].

Pediatric plastic surgery (burn surgery)

3D-printed transparent facemasks have been used in treating facial hypertrophic scars in young children with burns [94]. After 3D facial scanning and computer-aided design, the masks were printed in transparent biocompatible material, with medical-grade silicone gel added to provide extra pressure at the scar site. At one- and three-month follow-ups, average scar thickness had decreased, and facial appearance was satisfactory. Şenaylı et al. [95] reported their experience using 3D-printed splints to prevent contracture in 18 burned children. The study employed several materials, including polylactic acid (PLAFlex), polyurethane (PolyFlex), semiflexible copolyester (nGenFlex), and thermoplastic polyurethane (TPU). Splints were effective in 81.25% of upper extremities, 66.7% of lower extremities, and 100% of oral applications. Reported advantages included comfort, adequate fit, proper ventilation of tissue and skin, lightweight structure, and good aesthetics. Limitations included cost (equipment, software, labor for pre- and post-processing), long printing times, and the lack of comparative studies with conventional splints.

Pediatric gynecology

Pediatric straddle injury is the most common cause of genital trauma in girls under 14 years of age. Crain et al. [96] reported the use of a 3D-printed model to teach pediatric injury repair. They found that 3D vulvar models were cost-effective, easy to produce, and reusable. The models provided hands-on training for procedures that residents may not routinely encounter. The authors concluded that a 3D pediatric vulvar model can effectively simulate surgical experience and serve as a valuable teaching tool when combined with a didactic session on pediatric straddle injury.

Risk of bias

The overall methodological quality of the included studies was moderate. Most were descriptive studies, case reports, or case series, with inherent limitations such as small sample sizes, lack of control groups, and retrospective designs. Based on the Joanna Briggs Institute (JBI) checklists: (i) most case reports clearly described patient demographics, interventions, and outcomes but often lacked long-term follow-up or details on adverse events; (ii) case series frequently did not define inclusion criteria and showed variability in outcome reporting; and (iii) only a few studies incorporated any comparative analysis or controls.

Therefore, although the literature provides useful insights into the applications of 3D printing in pediatric surgery, the findings should be interpreted cautiously, given the predominance of low-level evidence and the risk of reporting and selection bias.

Discussion

Benefits/Advantages of 3D Printing

Simulation and trainee education: Simulation-based learning has proven its effectiveness in teaching various surgical disciplines, protecting patients, and reducing the risk of medical errors [45]. Following the principle of "never on a patient the first time," surgical educational programs have extensively incorporated simulation [39]. Indeed, the goal of simulation is to replicate the surgical environment as realistically as possible, both visually and tactilely. The various 3D printing technologies enable the creation of customized anatomical models, both normal and pathological, which can sometimes be reused. For these 3D-printed models to be beneficial in simulation-based learning, they must be realistic, easily reproducible, and cost-effective [59]. Furthermore, 3D printing techniques allow the creation of models depicting rare and complex pathologies, thus promoting education and learning in situations that are seldom encountered [84]. This is particularly valuable in the field of pediatric surgery, where a significant number of rare and complex pathologies are encountered. It is possible to create anatomical models of rare pathologies either by using real patient cases or by transforming a healthy case using computer-aided design techniques. This allows for the creation of a nearly infinite number of anatomical variations but exposes the risk of generating unrealistic or anatomically incorrect models, especially when developed by individuals without medical knowledge [67]. A close collaboration between engineers and physicians is, therefore, essential to avoid these pitfalls.

Surgical simulation sessions are sometimes conducted on animals or cadavers, which can raise ethical questions. 3D printing allows us to overcome the financial and ethical constraints of learning on live animals or cadavers [41,89].

Finally, 3D printing of anatomical models or simulators can be beneficial in countries where access to education and resources is limited. This technology offers a cost-effective and accessible means of providing medical education and surgical training, even in regions with limited access to traditional educational resources or expensive surgical simulation facilities [37].

Surgical planning: Several studies have highlighted the significance of 3D printing in surgical planning. Compared to 2D imaging, 3D anatomical models provide a volumetric representation of the surgical scene, just as it will be observed during the surgical procedure. By providing a 3D and detailed view of the patient's anatomy, 3D models enable surgeons to better understand the spatial relationships between structures, assess potential challenges, and plan the surgical approach accordingly. The application of 3D printing technology provides the possibility for individualized and precise treatment of patients. In the context of bone surgery, particularly in orthopedics or maxillofacial surgery, 3D printing enables the creation of customized cutting guides or the conformation of prostheses or osteosynthesis materials before the surgical procedure. This advanced technology facilitates the surgical intervention, streamlines the process, and saves valuable operating time. However, one might question the added value of 3D printing compared to digital 3D models. These digital models can be manipulated and analyzed on computer screens, providing valuable insights into the patient's condition and surgical approach. Indeed, in the clinical 3D modeling service integrated into routine pre-procedural care of pediatric patients with acquired or congenital heart disease reported at the Cardiac Center of The Children’s Hospital of Philadelphia, all cases were presented as printed models with flat-screen model viewing using a 3D PDF in the first year of the 3D modeling program. Over the following two years, there was increased utilization of new 3D visualization formats, such as computer-aided design-based modeling of potential surgical repairs and model viewing in virtual reality. There was an accompanying decrease in the use of printed models [72]. Using a digital model allows surgeons to explore and simulate various surgical scenarios without the limitations of physical objects. The integration of virtual reality technology enables immersive and interactive experiences, enabling surgeons to virtually navigate and manipulate the anatomical structures, test different surgical approaches, and evaluate potential outcomes in a dynamic and iterative manner.

Patient and family education: Patient and family education is a critical aspect of healthcare, ensuring that patients and their families are well-informed and empowered to actively participate in their care. Effective communication and clear explanations of medical conditions, treatment options, and potential risks are essential to help patients make informed decisions about their health. In the context of 3D printing, patient-specific 3D models can play a significant role in enhancing patient education and reducing parental anxiety [32-34]. These 3D models provide a visual representation of the patient's anatomy and the planned surgical approach, making complex medical concepts more accessible and understandable to patients and their families. For patients without a medical background, understanding 2D medical images can be challenging, as these images may appear abstract and difficult to interpret. In contrast, a 3D-printed model allows patients to see and touch a physical replica of their anatomy, which enhances their comprehension and engagement in the conversation.

Clinical use: 3D printing also has a direct clinical utility by enabling the creation of medical devices specific to a pathology or a patient. Implantable patient-specific devices are the next frontier of personalized medicine, positioned to improve the quality of care across multiple clinical disciplines [47,49]. However, development of patient-specific devices requires time and cost-effective processes to design, verify, and validate in adherence to the Food and Drug Administration (FDA) or other regulatory guidance for medical device manufacture [49]. The fabrication of personalized medical devices using 3D printing can be particularly valuable in children. Indeed, this makes it possible to create a device perfectly tailored to the size or morphology of each patient, which can be particularly valuable when a standard device does not exist or is not suitable for the patient. For children with congenital or traumatic upper limb amputation, prosthetic needs are complex due to their small size, rapid growth, and psychosocial development. Electric-powered units (i.e., myoelectric) and mechanical devices (i.e., body-powered) have been improved to accommodate children’s needs, but the cost of maintenance and replacement makes access difficult for many families. Advancements in computer-aided design and additive manufacturing offer the possibility of designing and printing transitional prostheses at a very low cost [24]. The development of custom-made implants represents the latest innovation in the field of limb salvage surgery after malignant bone tumors. Specifically, the sizes of the implants can be exactly proportioned to the sizes of the resected bone. This represents a significant advantage as compared with conventional prostheses and even modular ones. This custom design could be further improved with 3D-printed scaffolds. Indeed, 3D printing of scaffolds is a promising technique in the field of bone tissue engineering, allowing for the creation of custom scaffolds that precisely match the morphology of a patient’s bone defects. These 3D-printed scaffolds can be made from various materials, such as biopolymers, bioactive ceramics (like hydroxyapatite), and even composites that combine multiple materials. This technology can be used in various clinical applications, including the repair of critical bone defects, bone reconstruction after tumor surgery, and enhancing bone fusion in orthopedic procedures. While promising, 3D printing of scaffolds for bone tissue engineering presents challenges, particularly in terms of durability, controlled biodegradability, and the ability to replicate the mechanical properties of natural bone tissue. Furthermore, 3D-printed implants are useful in the reconstruction of bone segments with complex anatomy, in which conventional prostheses are not available, such as in the pelvis, tarsal bone, clavicle, or scapula [79].

In this context, 4D printing represents the future evolution of 3D printing, including an additional dimension, which is time. In 4D printing, the same process as 3D printing is used, but the printed objects possess the remarkable capability to dynamically transform their shape, properties, or functionality over time in response to external stimuli (like light, heat, humidity, or electric and magnetic fields). The success of 4D printing relies on the rational design of 4D printed objects, the selection of smart materials, and the availability of appropriate types of external stimuli. Thus, 4D printing enables the creation of medical devices that can adapt to growth or changes in tissues, which is particularly important in pediatric applications [47]. While 4D printing is expected to have a significant impact and promising prospects for practical applications in the near future, it is still in the development phase and presents several challenges, such as the complexity of precisely controlling transformations, the durability of materials in varied conditions, and production scaling. It is crucial to ensure that these new devices are safe, effective, and capable of reliably functioning in the complex environments of the human body.

Limitations/Barriers to 3D Printing

**Figure 5:**
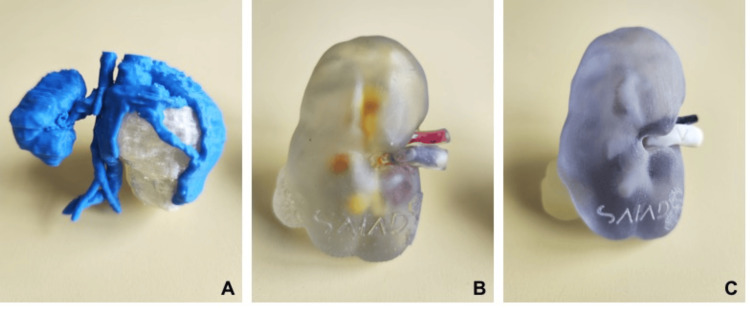
3D-printed models of kidneys with Wilms tumor using various 3D printing technologies: fused deposition modeling (A), stereolithography (B), and PolyJet (C). Note that realism decreases with low-cost models Image Credit: Yann Chaussy (with permission).

Realism of 3D-printed models: One of the main limitations of 3D printing currently concerns the realism of the 3D-printed objects [41]. It is quite easy to print rigid structures such as bone in a realistic manner, but this is much less true for soft tissues. It is thus much more challenging to reproduce the biomechanical properties of soft tissues with 3D-printed objects [92]. It is therefore essential to establish a cost-realism balance based on the level of significance one wishes to attribute to the 3D-printed object (Figure 5).

Thus, the 3D model should closely resemble reality in the context of simulation, which entails a certain material cost (material for printing, 3D printer) and human cost (for pre- and post-processing). Indeed, to enhance simulation performance, the 3D-printed model must be able to closely mimic the characteristics of the reproduced tissue in terms of haptic and tactile sensation. However, the 3D-printed model can be less realistic and more cost-effective if it is solely intended for improving information provided to the patient and their family or for educational purposes.

Quality of 3D printing: The quality of the 3D-printed model is directly dependent on the quality of the source image, which can be a limitation in children, where image acquisition may be performed with low-dose protocols to minimize radiation exposure (for CT scan) and where motion artifacts are more significant than in adults. The acquisition time in MRI may require general anesthesia to minimize these artifacts and obtain a sufficiently high-quality image for segmentation and 3D printing. In the first year of their 3D printing program, Ghosh et al. [72] reported that low-resolution or artifact-ridden imaging resulted in increased segmentation time and decreased quality of 3D-printed models [72]. It is, therefore, essential to establish appropriate imaging protocols when developing a 3D printing program.

Cost of 3D printing: As mentioned earlier, the cost of 3D printing can be an advantage, especially in the context of simulation, where it is possible to create and print low-cost simulators. However, it can be a barrier to creating realistic models of soft tissues. In addition to the cost of equipment (3D printers, software license fees), it is also necessary to consider the human cost involved in the pre- and post-processing phases. This also requires having competent individuals to carry out these specific tasks, highlighting the importance of the collaboration between physicians (who possess anatomical and medical knowledge) and engineers (who possess the technical skills for 3D printing). For example, Lemarteleur et al. [91] estimated their low-cost 3D-printed model of kidney less than $10 ($5 for reusable renal parenchyma and $0.30 for renal pelvis and ureter unit, with a printer costing $4,320), versus $100 for the molded silicone model and $400 for an anatomic model using jetting with hard colored multi-materials (with a printer costing $400,000).

Creation, development, maintenance, and funding of a 3D printing process: The creation of a patient-specific 3D-printed model involves a complex workflow consisting of several essential successive steps: image acquisition (CT, MRI, etc.), image processing and 3D virtual model creation (segmentation, registration, 3D reconstruction, smoothing, etc.), pre-print processes and the creation of files compatible with 3D printing (software selection), 3D printing (printer selection, material selection), post-processing, and evaluation of the final product. Each of these steps requires specific skills, not only in the medical field but also in computer science and biomedical engineering. As a result, establishing a 3D printing program relies on a multidisciplinary team that includes radiologists, clinical physicians, computer scientists, and biomedical engineers. However, since 3D printing in medical applications is a relatively specialized area, there is a shortage of trained personnel with the necessary skill sets. It is therefore essential to define the objectives of such a program in advance, including the expected uses, the required resources (both material and human), and the production costs. Recently, the establishment of in-house facilities for 3D modeling and printing at the point of care within hospitals has increased [77]. This approach allows the medical team to remain at the center of the production process, providing the flexibility to produce custom-made medical devices. However, it also necessitates close collaboration between doctors and engineers, a precise assessment of costs (infrastructure, equipment, personnel), and an objective evaluation of the outcomes.

Difficulty in highlighting the benefit of 3D-printed models: Although surgeons perceive a benefit from 3D models in the surgical planning phase, confirming it objectively remains challenging. As mentioned by Gosh et al., demonstrating the direct impact of 3D modeling on surgical outcomes is difficult due to the multifactorial nature of traditional postoperative metrics and difficulty performing a conclusive randomized trial in this setting [72]. It would, therefore, be necessary to conduct comparative studies of postoperative outcomes using exclusive 2D imaging, digital 3D models, and 3D printed models. Furthermore, there are other emerging 3D visualization modes such as virtual reality and augmented reality. For educational purposes, Awori et al. [69] found that participants endorsed a greater degree of understanding with VR models compared with 3D-printed models. However, these results were self-reported and such perceptions of learning impact are by nature subjective. Therefore, it will be necessary to conduct comparative studies to determine the role of 3D printing in various 3D visualization modes.

## Conclusions

Thanks to recent technological advances, 3D printing is increasingly applied across various areas of pediatric surgery. Studies highlight its role in simulation, trainee education, family counseling, and surgical planning. The use of 3D-printed implantable devices remains limited but is expected to expand with the growing emphasis on personalized medicine. Current challenges include the realism of printed models, costs, and the difficulty of clearly demonstrating clinical benefits. Well-designed comparative studies will be needed to objectively establish the value of 3D printing in pediatric surgery.

## Appendices

**Table 3:** Included articles and their study characteristics

Reference	Authors	Year	Study design	Field
[1]	Wickramasinghe et al.	2018	Case series/descriptive study	Orthopedics
[2]	Caffrey et al.	2019	Simulation-based comparative study	Orthopedics
[3]	Neijhoft et al.	2022	Descriptive simulation study	Orthopedics
[4]	Hedelin et al.	2019	Technical report (validation study)	Orthopedics
[5]	Cao et al.	2022	Retrospective comparative cohort study	Orthopedics
[6]	Facco et al.	2022	Case series/descriptive study	Orthopedics
[7]	Bovid et al.	2019	Case report with simulation planning	Orthopedics
[8]	Miao et al.	2024	Case series with technical innovation	Orthopedics
[9]	Burzyńska et al.	2016	Pilot study (technical/experimental)	Orthopedics
[10]	Zheng et al.	2017	Case-control comparative study	Orthopedics
[11]	Sun et al.	2023	Retrospective comparative study	Orthopedics
[12]	Liu et al.	2022	Retrospective comparative study	Orthopedics
[13]	Wan et al.	2021	Case report	Orthopedics
[14]	Hu et al.	2020	Case series	Orthopedics
[15]	Alessandri et al.	2022	Case report	Orthopedics
[16]	Cai et al.	2023	Technical report/case series	Orthopedics
[17]	Qu et al.	2023	Technical note/case series	Orthopedics
[18]	Dow et al.	2022	Case report	Orthopedics
[19]	Mohammadi et al.	2020	Technical note/feasibility study	Orthopedics
[20]	Ccorimanya et al.	2019	Technical report/case application	Orthopedics
[21]	Gretsch et al.	2016	Technical development study	Orthopedics
[22]	Zuniga et al.	2018	Prospective observational study	Orthopedics
[23]	Zuniga et al.	2015	Technical note/pilot study	Orthopedics
[24]	Zuniga et al.	2019	Functional evaluation study	Orthopedics
[25]	Bhat et al.	2021	Prospective observational study	Orthopedics
[26]	Zuniga et al.	2019	Technical protocol	Orthopedics
[27]	Cabibihan et al.	2020	Feasibility study	Orthopedics
[28]	Lazzeri et al.	2022	Observational study	Orthopedics
[29]	Katt et al.	2021	Case reports	Orthopedics
[30]	Elbaum	2020	Descriptive study	Orthopedics
[31]	Wojciechowski et al.	2022	Case study	Orthopedics
[32]	Lioufas et al.	2016	Simulation study/educational model	Head and neck surgery
[33]	Chou et al.	2017	Prospective survey study	Head and neck surgery
[34]	Jimenez et al.	2023	Prospective observational study	Head and neck surgery
[35]	Longfield et al.	2015	Technical report/simulation tool	Head and neck surgery
[36]	Freiser et al.	2021	Technical development study	Head and neck surgery
[37]	Barber et al.	2016	Simulation study	Head and neck surgery
[38]	Kavanagh et al.	2017	Technical note	Head and neck surgery
[39]	Chebib et al.	2022	Technical development and evaluation study	Head and neck surgery
[40]	Reighard et al.	2019	Technical development study	Head and neck surgery
[41]	Falls et al.	2022	Comparative simulation study	Head and neck surgery
[42]	Michaels et al.	2022	Descriptive educational study	Head and neck surgery
[43]	Chang et al.	2020	Simulation study	Head and neck surgery
[44]	Lee et al.	2019	Case report	Head and neck surgery
[45]	Rose et al.	2015	Simulation study	Head and neck surgery
[46]	Arcieri et al.	2018	Case report	Head and neck surgery
[47]	Morrison et al.	2015	Prospective case series	Head and neck surgery
[48]	Les et al.	2019	Prospective cohort study	Head and neck surgery
[49]	Ramaraju et al.	2022	Technical protocol	Head and neck surgery
[50]	Wang and Qi	2020	Case report	Head and neck surgery
[51]	Akiki et al.	2021	Case report	Head and neck surgery
[52]	Chai et al.	2021	Simulation study	Head and neck surgery
[53]	Arunkumar et al.	2023	Case series	Head and neck surgery
[54]	Wu and Lv	2023	Case report	Head and neck surgery
[55]	Thurzo et al.	2022	Case report	Head and neck surgery
[56]	Groot et al.	2023	Retrospective cohort study	Head and neck surgery
[57]	Di-Luciano et al.	2022	Case series	Head and neck surgery
[58]	London et al.	2021	Simulation model validation	Neurosurgery/spinal surgery
[59]	Weinstock et al.	2017	Development study/simulation tool	Neurosurgery/spinal surgery
[60]	González-López et al.	2023	Simulation study	Neurosurgery/spinal surgery
[61]	Graffeo et al.	2023	Technical note/simulation series	Neurosurgery/spinal surgery
[62]	Chacko et al.	2023	Validation study (model fidelity)	Neurosurgery/spinal surgery
[63]	Weinstock et al.	2015	Case series	Neurosurgery/spinal surgery
[64]	Cao et al.	2021	Retrospective comparative study	Neurosurgery/spinal surgery
[65]	Vissarionov et al.	2020	Comparative study	Neurosurgery/spinal surgery
[66]	Lou et al.	2022	Technical report/observational study	Neurosurgery/spinal surgery
[67]	Hopfner et al.	2021	Technical study/simulation	Cardiac and vascular surgery
[68]	Brunner et al.	2022	Simulation/educational tool study	Cardiac and vascular surgery
[69]	Awori et al.	2023	Comparative effectiveness study	Cardiac and vascular surgery
[70]	Karsenty et al.	2022	Prospective observational study	Cardiac and vascular surgery
[71]	Pizzuto et al.	2023	Case report	Cardiac and vascular surgery
[72]	Ghosh et al.	2022	Retrospective cohort study	Cardiac and vascular surgery
[73]	Ryan et al.	2018	Retrospective comparative study	Cardiac and vascular surgery
[74]	Parthasarathy et al.	2021	Validation/feasibility study	Cardiac and vascular surgery
[75]	Riggs et al.	2018	Case report	Oncological surgery
[76]	Jug	2021	Case report	Oncological surgery
[77]	Parthasarathy et al.	2023	Technical process description	Oncological surgery
[78]	Youn et al.	2023	Prospective observational study	Oncological surgery
[79]	Girón-Vallejo et al.	2018	Case report	Oncological surgery
[80]	Sánchez-Sánchez et al.	2018	Retrospective cohort study	Oncological surgery
[81]	Beltrami et al.	2021	Case series	Oncological surgery
[82]	Gong et al.	2023	Case series	Oncological surgery
[83]	Zhu et al.	2021	Case report	Oncological surgery
[84]	Burdall et al.	2016	Simulation study	Digestive surgery
[85]	Williams et al.	2018	Simulation study	Digestive surgery
[86]	Park et al.	2022	Case report/technical feasibility	Digestive surgery
[87]	Krois et al.	2022	Simulation study	Digestive surgery
[88]	Hong et al.	2021	Technical simulation study	Thoracic surgery
[89]	Matsuo et al.	2018	Case report	Thoracic surgery
[90]	Duan et al.	2022	Technical design study	Thoracic surgery
[91]	Lemarteleur et al.	2021	Technical development and survey	Urology
[92]	Chandak et al.	2019	Case series	Urology
[93]	He et al.	2022	Case report	Urology
[94]	Wei et al.	2017	Prospective interventional study	Plastic surgery/burns
[95]	Şenaylı et al.	2021	Prospective clinical study	Plastic surgery/burns
[96]	Crain et al.	2021	Educational simulation study	Gynecology
